# OSTEOARTHRITIS AND DEPRESSION IN A MALE VA POPULATION

**DOI:** 10.1093/geroni/igz038.591

**Published:** 2019-11-08

**Authors:** Christopher J Burant, Gregory Graham, Denise M Kresevic, Gary Deimling, Said Ibrahim, Kent Kwoh

**Affiliations:** 1 Case Western Reserve University, Cleveland, Ohio, United States; 2 Frances Payne Bolton School of Nursing , Case Western Reserve University, Cleveland, Ohio, United States; 3 Northeast Ohio VAMC, Cleveland, Ohio, United States; 4 Weill Cornell Medicine, New York, New York, United States; 5 University of Arizona Arthritis Center, Tucson, Arizona, United States

## Abstract

Osteoarthritis (OA) is a leading cause of disability among older adults. By 2050, approximately 60 million will suffer from arthritis adding up to a total societal cost of $65 billion. Chronic illnesses resulting in pain, and functional decline have been associated with depression in previous studies. The primary goal of this study is to investigate whether OA severity, as measured by the Western Ontario McMasters Arthritis Composite (WOMAC), impacts reported levels of depression and to what degree clinical and sociodemographic variables play a part. A causal model was developed and tested examining the antecedents of OA disease severity and depression. Information on clinical, demographic, socioeconomic, and psychosocial variables was collected on 596 male Veterans with moderate to severe symptomatic OA of the knee\hip. A Confirmatory Factor Analysis was conducted to determine the factor structure of the WOMAC. A 2nd order three factor solution (pain, stiffness, and function) fit the data well (TLI of .94, a CFI of .94 and a RMSEA of .058). The results of the Structural Equation Model reveal a final model that fit the data well (TLI of .95, a CFI of .97 and a RMSEA of .047). Depression was predicted by higher WOMAC scores (beta=.37 , p<.01); higher levels of comorbidity (beta= .11, p<.05); younger age (beta= -.29, p<.01); being white (beta=-.11, p<.05); lower levels of income (beta= -.12, p<.05); lower levels of religiosity (beta= 11, p<.05). Clinicians should be aware of the impact of disease severity when treating OA patients with depression.

